# Seasonal variation in soil algal community structure in different forest plantations in subtropic China

**DOI:** 10.3389/fpls.2023.1181184

**Published:** 2023-07-13

**Authors:** Liman Wei, Qiong Zhao, Xiangyu Chen, Qingye Sun, Xiang Zhang, Yongjing Chen

**Affiliations:** ^1^ School of Resources and Environmental Engineering, Anhui University, Hefei, Anhui, China; ^2^ Agricultural Sensors and Intelligent Perception Technology Innovation Center of Anhui Province, Zhongke Hefei Institutes of Collaborative Research and Innovation for Intelligent Agriculture, Hefei, China; ^3^ Institute of Intelligent Machines, Hefei Institutes of Physical Science, Chinese Academy of Sciences, Hefei, China

**Keywords:** gene sequencing, forest plantations, algal community structure, environmental factors, seasonal variations

## Abstract

Algae exert great impact on soil formation and biogeochemical cycling. However, there is no full understanding of the response of soil algal community structure to the seasonal fluctuations in temperature and moisture and changes of soil physicochemical properties across different forests. Here, based on 23S rRNA gene sequencing, we analyzed soil algal community structure in four different forest plantations in two seasons and examined soil physiochemical properties. The results showed the significantly seasonal variation in soil algal community structure, with the higher overall diversity in summer than in winter. In addition, there existed significant correlations between soil algae (species composition, relative abundance, diversity index) and physicochemical properties (pH, total phosphorus, organic matter and nitrate nitrogen), suggesting that edaphic characteristics are also largely responsible for the variation in soil algal community. Nevertheless, the seasonal variation in algal community structure was greater than the variation across different forest plantations. This suggest temperature and moisture are more important than soil physicochemical properties in determining soil algal community structure. The findings of the present study enhance our understanding of the algal communities in forest ecosystems and are of great significance for the management and protection of algal ecosystem.

## 1 Introduction

Algae occur in nearly all terrestrial ecosystems on earth (Metting, 1981; Zancan et al., 2006; Lichner et al., 2013). They are pioneering organisms in soil formation processes (Patova et al., 2016; Marques et al., 2017; Agnelli et al., 2021). As an indispensable component of microflora, soil algae interact with the environment to advance the soil formation (Yang et al., 2016; Maltsev et al., 2017a; Maltsev et al., 2017b; Abinandan et al., 2019). The principal function of soil algal communities in promote soil formation and biochemical processes include dinitrogen fixation, stabilization of aggregates, mineralization of organic matter, elevate soil air and water retention capacity, improve soil microbial activity and structure, et al. (Metting, 1981; Glaser et al., 2018; Alvarez et al., 2021). Thereafter, algae play an important role in soil and vegetation restoration in waste rock dumps and anthropogenically disturbed lands (Carvalhido et al., 2021; Oliveira and Maciel-Silva, 2022). Making clear soil algal community composition and its influencing factors in forest ecosystems is of great significance for better understanding their ecological functions.

Algal community structure was influenced by both biotic and abiotic factors (Nisha et al., 2007; Kostryukova et al., 2021; Sawestri and Rais, 2021; Rahman, 2022). Recent studies have documented that soil pH, moisture content, and nutrients control the algal community structure (Bohlen et al., 2001; Prasanna, 2007; Zhang et al., 2021; Zhao et al., 2022; Gabriel et al., 2023; Graham and Knelman, 2023). The soil algal community structure is affected by the environment may be because different taxonomic groups prefer to different soil pH and nutrient conditions (Baldrian et al., 2012). For instance, Cyanobacteria tend to grow in neutral and alkaline environment, while acidic soil is more suitable for Chlorophyta growth (Bailey et al., 2010). Across large spatial scales, algal community are strongly impacted by regional climate, altitude and light intensity. Novakovskaya (Novakovskaya et al., 2020) pointed that taxonomic diversity of algae decreases along the altitude gradient from mountain meadow to mountain tundra. Dirborne (Dirborne and Ramanujam, 2017) found that soil in undisturbed broadleaf forest supported more diverse algal species than pine forest. Such studies facilitate the understanding of the responses of algal communities to environmental changes. However, how seasonal variation in temperature and moisture interact with soil physiochemistry properties to affect soil algal community structure is still poor known.

The traditional method for identifying algae is microscopic observation. For soil samples, algae should be cultured before microscopic observation. However, some algal groups can not be cultured due to the preference of the culture medium (Amann et al., 1995; Novakovskaya et al., 2020). Nowadays, molecular sequencing was applied to identify the algal community structure instead of microscopic observation (Satjarak et al., 2020). The DNA bands can be used to directly compare the base pairs (Sherwood et al., 2008; Jiang et al., 2017; Yan et al., 2020). This method can avoid errors during purification, culture process and identification (Rippin et al., 2018; Zhu et al., 2018; Mikhailyuk et al., 2019).

The objectives of this study were to: (1) Investigate whether there were significant seasonal differences in the diversity index and composition of soil algae in different subtropic forest plantations; (2) Compare the relative important of effects of seasonal variation in temperature and moisture and changes of soil physiochemistry properties on algal community structure, and reveal their potential relationship. To achieve these goals, samples of soils and algae were collected from four typical forest plantations (*Liquidambar formosana*, *Cyclobalanopsis glauca*, *Pinus massoniana* and *Cunninghamia lanceolata*) in both winter and summer in a forest farm in subtropical China. This work can advance our understanding of the responses of algal community to environmental changes.

## 2 Materials and methods

### 2.1 Study area

**Figure 1:**
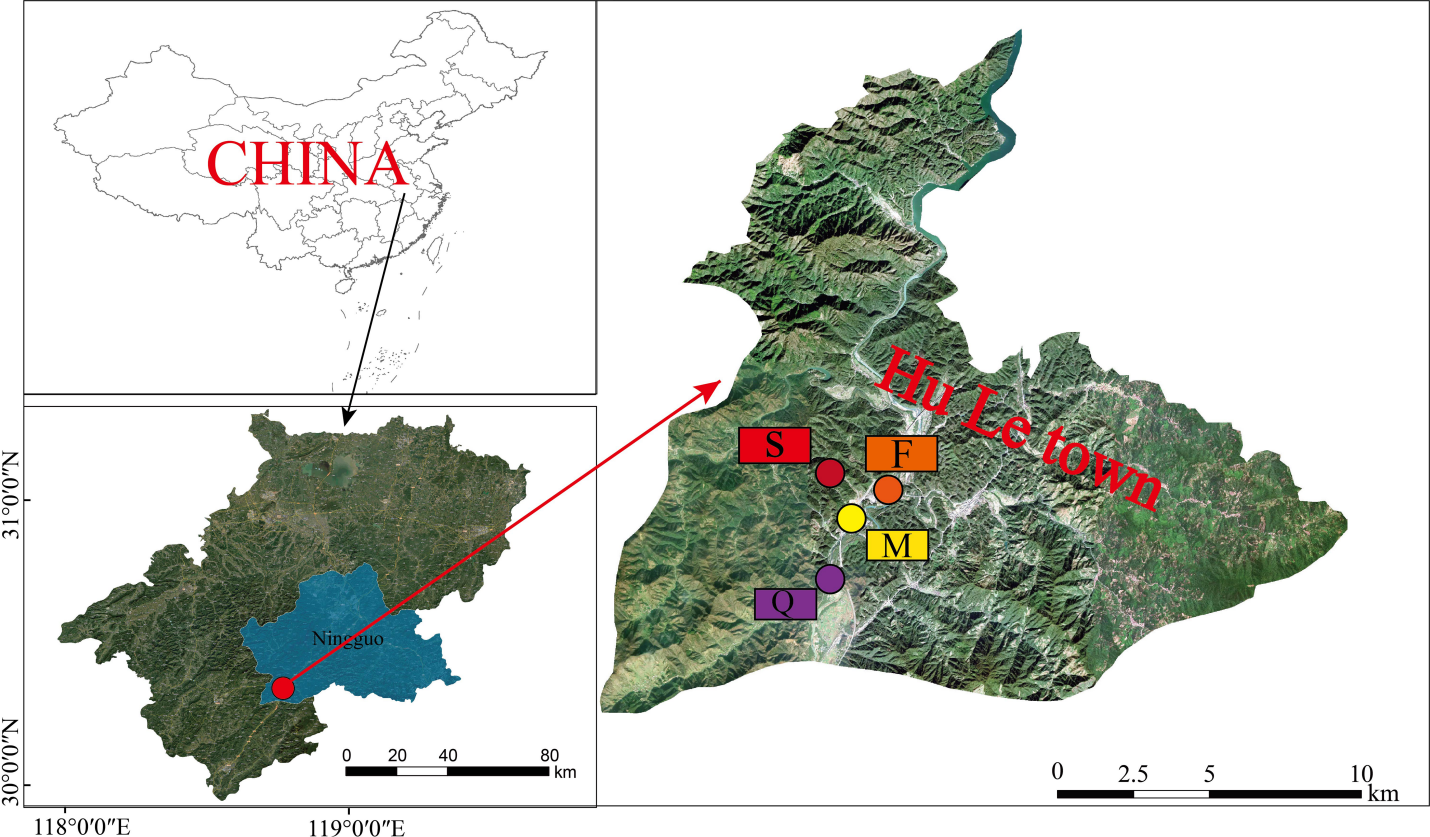
Location of Hule Forest Farm in Anhui of China and the sampling plots. (F, *Liquidambar formosana*; Q, *Cyclobalanopsis glauca*; M, *Pinus massoniana*; S, *Cunninghamia lanceolata*).

This study was carried out in the Hule Forest Farm (30°18’52”-30°20’55” N, 118°45’6”-118°45′54″ E) in Ningguo city, Anhui Province, China (**Figure 1**). It belongs to the hilly area of the southern Anhui Province, and has a subtropical monsoon climate, with an annual average temperature of 15.4°C and annual average precipitation of 1426.9 mm. The annual frost-free period is 226 d, the sunshine duration is 2038 h, and the average wind speed is 2.1 m/s (Wei et al., 2023). The main tree species in this forest farm were *Liquidambar formosana*, *Cyclobalanopsis glauca*, *Pinus massoniana* and *Cunninghamia lanceolata*.

In December 2020, we selected four typical forest plantations in the farm as our study sites. The four plantations are 21-year-old pure *Liquidambar formosana*, 66-year-old pure *Cyclobalanopsis glauca*, 64-year-old pure *Pinus massoniana* and 40-year-old pure *Cunninghamia lanceolata*.

### 2.2 Sample collection

Samples were collected on December 2020 and June 2021. Three quadrats (20 m × 20 m) were established for sampling in each forest plantation. The surface soil (0 - 5 cm) was collected from four corners and center of each quadrat taken with a sterile scraper, mixed evenly, put into sealed bags, and brought back to the laboratory in a cool box for algal community structure analysis (Ye, 1983). The winter and summer samples were designated as the “Win” and “Sum” groups, respectively. Win-LF, *Liquidambar formosana* plantation in winter; Win-CG, *Cyclobalanopsis glauca* plantation in winter; Win-PM, *Pinus massoniana* plantation in winter; Win-CL, *Cunninghamia lanceolata* plantation in winter; Sum-LF, *Liquidambar formosana* plantation in summer; Sum-CG, *Cyclobalanopsis glauca* plantation in summer; Sum-PM, *Pinus massoniana* plantation in summer; Sum-CL, *Cunninghamia lanceolata* plantation in summer.

### 2.3 Physicochemical analyses

Soil pH was measured with a glass electrode (1:2.5 soil:water ratio) (Wan et al., 2014). Soil moisture content (moisture percentage based on natural wet soil) is determined by drying method. Soil organic matter (OM) was determined by K_2_Cr_2_O_7_-H_2_SO_4_ oxidation method (Nelson and Sommers, 1996). Concentration of soil total nitrogen (TN) and phosphorus (TP) was determined on a continuous-flow autoanalyzer (AutoAnalyzer 3, Bran + Luebbe GmbH, Germany) after the soil was digested in concentrated H_2_SO_4_ with a catalyst (mixture of CuSO_4_ and K_2_SO_4_) (Lu, 1999). Concentrations of ammonia nitrogen (NH_4_^+^−N) and nitrate nitrogen (NO_3_^-^− N) were analyzed colorimetrically on the autoanalyzer after the field moist soil was extracted with 2 mol L^-1^ KCl (Weatherburn, 1967; Doane and Horwáth, 2003). Concentration of soil alkali-hydrolyzed nitrogen (AN) was determined by diffusion absorption method (Science, 1978). Soil available phosphorus (AP) was extracted with Mehlich 3 extractant and determined by molybdenum blue colorimetric method (Mehlich, 2008). Concentration of soil available potassium (AK) was extracted with ammonium acetate solution (pH = 7.0) and determined by flame photometry (Science, 1978).

### 2.4 DNA extraction, PCR amplification, and illumina sequencing

DNA extraction was conducted using the method described by Su (Su et al., 2006; Carrigg et al., 2007). R.Sherwood demonstrated the feasibility of using 23S rRNA universal primers for amplification and sequencing of this plastid marker for multiple eukaryotic algal and Cyanobacterial groups (Sherwood and Presting, 2007). PCR amplification of the algal 23S rRNA genes was performed using the forward primer p23rv_f1 (5’-GGACAGAAAGACCCTATGAA-3’) and the reverse primer p23rv_r1 (5’-TCAGCCTGTTATCCCTAGAG -3’). The PCR components contain 5 μL of buffer (5 ×), 0.25 μL of Fast pfu DNA Polymerase (5 U/μL), 2 μL (2.5 mM) of dNTPs, 1 μL (10 μM) of each Forward and Reverse primer, 1 μL of DNA template, and 14.75 μL of ddH_2_O. Thermal cycling consist of initial denaturation at 94°C for 2 min, followed by 35 cycles consisting of denaturation at 94°C for 20 s, annealing at 55°C for 30 s, and extension at 72°C for 30 s, with a final extension of 10 min at 72°C. PCR amplicons were purified with Vazyme VAHTSTM DNA Clean Beads (Vazyme, Nanjing, China) and quantified using the Quant-iT PicoGreen dsDNA Assay Kit (Invitrogen, Carlsbad, CA, USA). After the individual quantification step, amplicons were pooled in equal amounts, and pair-end sequencing was performed using the Illlumina MiSeq platform at Shanghai Personal Biotechnology Co., Ltd (Shanghai, China).

### 2.5 Sequencing data processing

Illumina technology was used to double-end sequencing, quality filtering, denoising and merging of DNA sequences. QIIME2 and R package (V3.2.0) were used to analyze the sequence data. The ASV table was extracted and the α diversity index of ASV level was calculated.

### 2.6 Statistical analysis

Physicochemical variables were displayed as the mean value ± standard error (SE) in Excel 2016 (Microsoft Office 2016, Microsoft, USA). Statistical analysis was performed through single factor analysis of variance (ANOVA), and Duncan’s multiple range test (P < 0.05) was utilized for statistical significance analysis. Heatmap were draw in the R (4.0.2). The relationship between the algal community and environmental factor was determined by redundancy analysis (RDA) using RStudio (version 1.2.1335) with the vegan package. Linear discriminant analysis (LDA) effect Size (LEfSe) was used to identify algal taxa with significant differences among different forest plantations, performed by an online platform for data analysis (https://www.omicstudio.cn).

## 3 Results

### 3.1 Soil physicochemical parameters

All of the soil samples were acidic with the pH values ranged from 5.03 to 6.43. The soil moisture content of the four forest plantations in summer (25.01% − 32.81%) was higher than that in winter (18.38% - 19.60%). In general, concentrations of all inorganic nitrogen (NH_4_^+^−N, NO_3_^-^−N, AN) and TN were higher in summer than winter in all plantations. The concentrations of TP and AK were significantly higher in summer than winter while the AP was higher in winter than summer for most forest plantations. There was no significant seasonal variation in OM concentration in all plots (**Table 1**).

**Table 1:** Soil physicochemical properties of four forest plantations in summer and winter.

Physicochemical properties	Seasons	*Liquidambar formosana*	*Cyclobalanopsis glauca*	*Pinus* *massoniana*	*Cunninghamia lanceolata*
Moisture content (%)	Winter	19.60 ± 0.24^b^	24.11 ± 0.86^a^	18.38 ± 0.80^b^	20.10 ± 0.58^b^
	Summer	32.81 ± 0.51^a^	25.01 ± 1.39^b^	22.64 ± 0.28^b^	31.82 ± 1.00^a^
pH	Winter	5.88 ± 0.13^a^	5.03 ± 0.01^c^	5.35 ± 0.08^b^	5.12 ± 0.06b^c^
	Summer	6.43 ± 0.06^a^	5.29 ± 0.04^c^	5.58 ± 0.13^b^	5.51 ± 0.04^bc^
NH_4_^+^−N (mg kg^-1^)	Winter	4.92 ± 0.26^b^	9.09 ± 0.12^a^	7.47 ± 0.80^a^	4.63 ± 0.87^b^
	Summer	8.13 ± 0.24^b^	12.30 ± 0.47^a^	8.47 ± 0.71^b^	6.75 ± 0.87^b^
NO_3_^-^−N (mg kg^-1^)	Winter	2.81 ± 0.18^c^	6.16 ± 0.34^a^	2.89 ± 0.37^c^	4.97 ± 0.15^b^
	Summer	3.69 ± 0.15^c^	9.06 ± 0.18^a^	5.06 ± 0.51^b^	8.45 ± 0.24^a^
AN (mg Kg^-1^)	Winter	101.20 ± 2.70^b^	136.80 ± 8.40^a^	36.30 ± 1.90^c^	51.40 ± 1.20^c^
	Summer	147.90 ± 14.70^b^	197.30 ± 4.10^a^	71.20 ± 6.40^d^	106.10 ± 6.20^c^
TN (g Kg^-1^)	Winter	16.50 ± 0.40^a^	19.40 ± 0.30^a^	17.10 ± 2.20^a^	15.20 ± 0.20^a^
	Summer	9.80 ± 0.80^b^	23.70 ± 2.50^a^	18.10 ± 3.10^ab^	20.10 ± 3.00^a^
AP (mg kg^-1^)	Winter	4.94 ± 0.08^c^	9.64 ± 0.13^b^	13.94 ± 0.03^a^	9.24 ± 0.18^b^
	Summer	2.74 ± 0.13^d^	7.93 ± 0.11^b^	14.40 ± 0.51^a^	6.25 ± 0.50^c^
TP (g Kg^-1^)	Winter	1.20 ± 0.00^b^	1.20 ± 0.00^b^	2.20± 0.20^a^	1.20 ± 0.10^b^
	Summer	1.60± 0.10^b^	1.60 ± 0.10^b^	1.80 ± 0.10^b^	3.20 ± 0.20^a^
AK (mg Kg^-1^)	Winter	131.50 ± 2.20^a^	109.10 ± 0.40^c^	133.80 ± 3.30^a^	117.90 ± 3.00^b^
	Summer	86.80 ± 7.00^b^	147.20 ± 8.90^a^	148.60 ± 5.20^a^	154.10 ± 6.50^a^
OM (g Kg^-1^)	Winter	22.80 ± 0.50^c^	37.00 ± 0.80^a^	30.90 ± 0.40^b^	19.60 ± 1.20^d^
	Summer	22.60 ± 0.30^c^	37.20 ± 0.70^a^	29.40 ± 0.70^b^	20.30 ± 0.60^d^

Values are means ± standard error (SE), n = 3. Different letters within each line indicate significant differences among the four forest plantations (p < 0.05).

### 3.2 Diversity of the soil algal community

**Figure 2:**
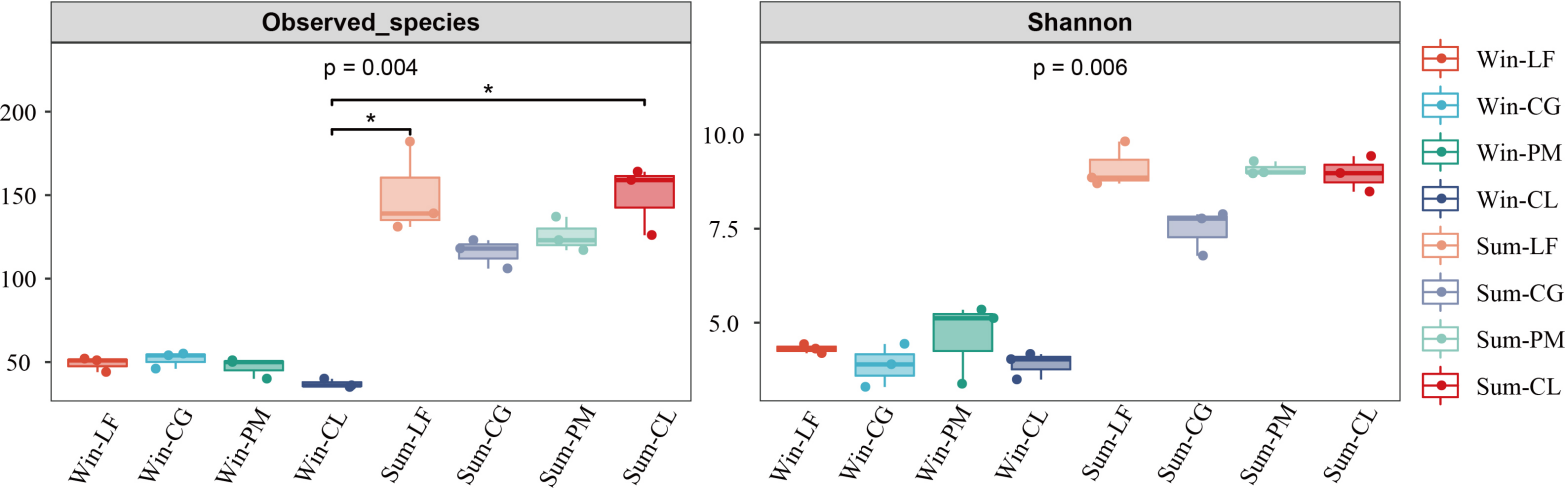
The diversity index of soil algal communities in four forest plantations in summer and winter. Win-LF, *Liquidambar formosana* plantation in winter; Win-CG, *Cyclobalanopsis glauca* plantation in winter; Win-PM, *Pinus massoniana* plantation in winter; Win-CL, *Cunninghamia lanceolata* plantation in winter; Sum-LF, *Liquidambar formosana* plantation in summer; Sum-CG, *Cyclobalanopsis glauca* plantation in summer; Sum-PM, *Pinus massoniana* plantation in summer; Sum-CL, *Cunninghamia lanceolata* plantation in summer.

**Figure 3:**
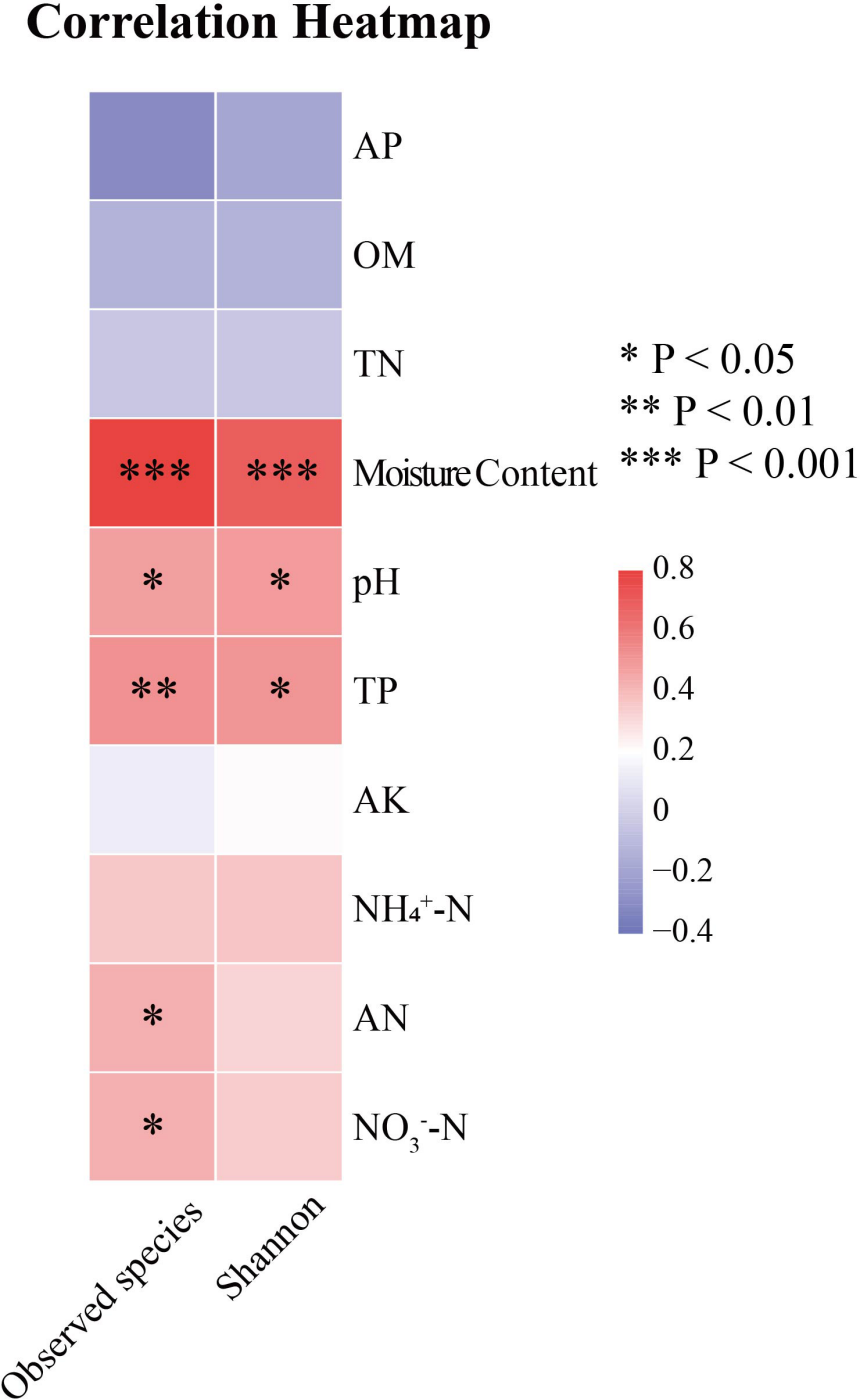
Correlation analysis of diversity index and environmental factors.

The results of diversity indices showed that the observed species (*p* < 0.005) and Shannon index (*p* < 0.01) of the soil algae differed significantly between winter and summer (**Figure 2**). According to the spearman’s correlation analyses, soil moisture content (*p* < 0.001), pH (*p* < 0.05) and TP (*p* < 0.05) were positively and significantly correlated with observed species and Shannon index. Furthermore, AN and NO_3_^-^−N were significantly and positively correlated with observed species (*p* < 0.05) (**Figure 3**).

### 3.3 Soil algal community composition

We obtained a total of 724814 high-quality valid sequences of 23S rRNA gene in all 24 samples. We clustered the sequences based on 100% sequence similarity and obtained 19017 ASVs (**Supplementary Files**). A total of 11 phyla, 44 classes, 89 orders, 145 families and 205 genera were identified.

**Figure 4:**
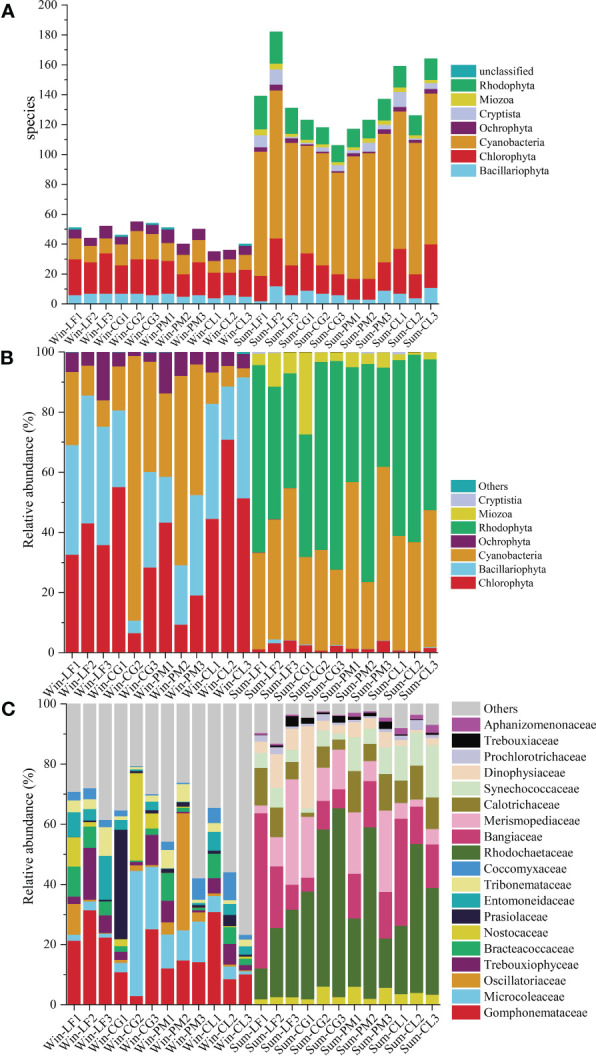
Algal community composition of soil sample in Hule Forest farm **(A)** Number of species; **(B)** Phylum level; **(C)** Family level.

There were significant seasonal differences in soil algal community structure, and the overall richness of soil algae was higher in summer than that in winter (**Figure 4A**). In winter, 82 species of soil algae were identified, belonging to 5 phyla, 18 classes, 33 orders, 43 families, 46 genera. The richness of soil algae in winter was highest in *Cyclobalanopsis glauca* plantation, and lowest in *Cunninghamia lanceolata* plantation. In summer, we identified 294 species of algae, belonging to 7 phyla, 40 classes, 82 orders, 132 families, 193 genera. The richness of soil algae in summer was highest in *Liquidambar formosana* plantation, and lowest in *Cyclobalanopsis glauca* plantation. Chlorophyta and Cyanobacteria were the most abundant phyla in both winter and summer, accounting for 37.80% and 48.00% of the total species, respectively.

The relative abundance of each phylum of soil algae in four forest plantations changed significantly in different seasons (**Figure 4B**). In winter, Chlorophyta (36.71%) was the most abundant phylum, then decreased in the order of Bacillariophyta (28.70%), Cyanobacteria (28.07%) and Ochrophyta (6.46%). The most abundant phylum in summer was Rhodophyta (52.66%), followed by Cyanobacteria (38.81%), Miozoa (6.15%) and Chlorophyta (1.98%).

Analysis of the 10 most abundant family in each group revealed significant variations in the soil algal community composition across seasons and plantations (**Figure 4C**). The dominant families differed greatly between the two seasons. In winter, the dominant families were Gomphonemataceae, Microcoleaceae, Oscillatoriaceae, Trebouxiophyceae, Bracteacoccaceae, Nostocaceae, Prasiolaceae, Entomoneidaceae, Tribonemataceae and Coccomyxaceae. And the abundance of Microcoleaceae (21.82%) and Nostocaceae (12.15%) in *Cyclobalanopsis glauca* plantation was significantly higher than that in other three plantations. The abundance of Oscillatoriaceae (15.36%) in *Pinus massoniana* plantation was significantly higher than that in other three plantations. In summer, the dominant families were Rhodochaetaceae, Bangiaceae, Merismopediaceae, Calotrichaceae, Synechococcaceae, Dinophysiaceae, Prochlorotrichaceae, Trebouxiaceae, Nostocaceaeand Aphanizomenonaceae. The abundance of Rhodochaetaceae (50.29%) in *Cyclobalanopsis glauca* plantation was significantly higher than that in the other three plantations.

**Figure 5:**
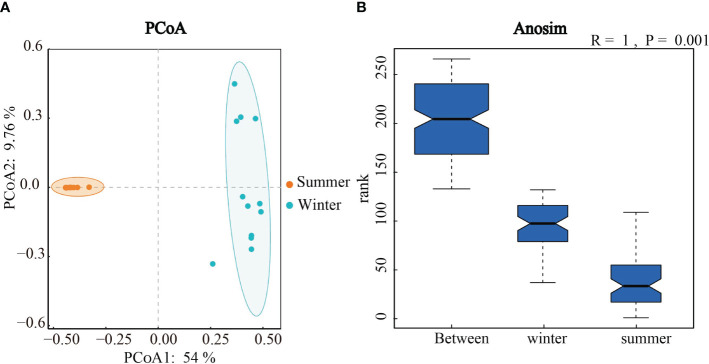
Principal co-ordinates analysis **(A)** and Anosim test **(B)** of algal Community structure in winter and summer.

In order to explore seasonal differences in community structure of soil algae, principal co-ordinates analysis (PCoA) and Anosim test were used (**Figure 5**). The Anosim analyses showed the significant seasonal differences in the algal community structure in Hule Forest Farm (*p* = 0.001) (**Figure 5B**). Similarly, the PCoA showed that the algal communities in summer and winter were well separated on the two axes, which means the seasonal variation in algal community structure was greater than the variation across different forest plantationsthe (**Figure 5A**).

**Figure 6:**
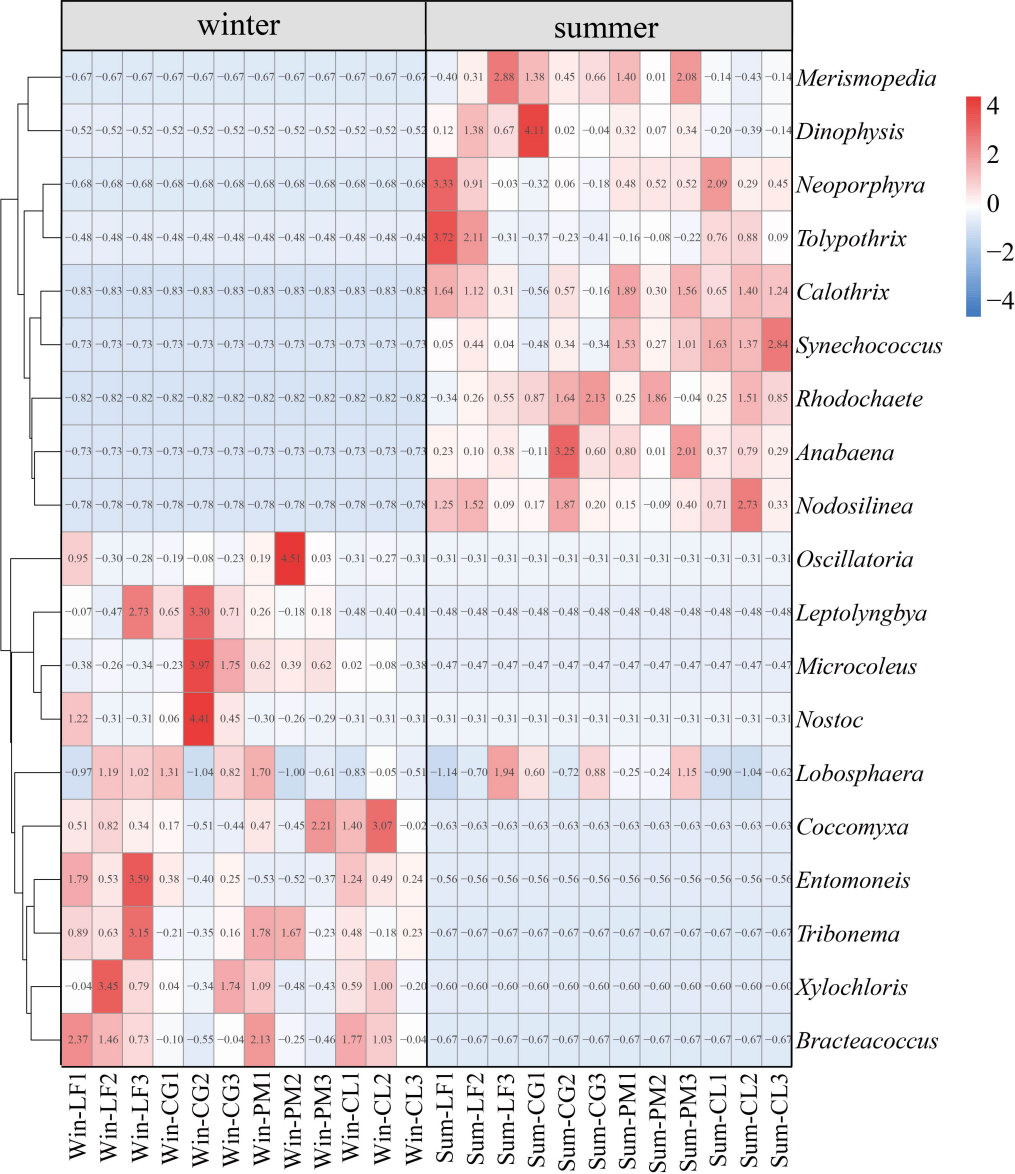
Heat map depicting the hierarchy cluster results for the abundance of algae at the genus level. Ten most abundant genera of each season are shown. Red indicates high relative abundance and blue indicates low relative abundance.

Heat map analysis can reflect the cluster analysis of soil algal community structure and the relative abundance of each community composition of four forest plantations in different seasons. The results of heat map analysis of soil algal community structure were the same as those of PCoA (**Figure 5A**), and there were significant seasonal differences in soil algal community structure. Analysis of the 10 most abundant genera in each group revealed characteristic changes in soil algal community composition across seasons and forest types (**Figure 6**). In winter, *Microcoleus* was abundant in *Liquidambar formosana* plantations, *Cyclobalanopsis glauca* plantations and *Pinus massoniana* plantations, but rarely distributed in *Cunninghamia lanceolata* plantations. *Leptolyngbya* did not appear in *Cunninghamia lanceolata* plantations. *Oscillatoria* (relative abundance < 0.3%) and *Nostoc* (relative abundance < 0.1%) were very rare in *Cunninghamia lanceolata* plantations. In addition, the relative abundance of *Entomoneis* (8.89%) in Win-LF group was significantly higher than that in the other three forests (Win-CG: 2.24%; Win-PM: 0.31%; Win-CL: 4.26%). *Coccomyxa*, *Tribonema*, *Xylochloris* and *Bracteacoccus* were present in all four forests. In summer, the 10 dominant genera were present in all four forests. The relative abundance of *Neoporphyra* in Sum-CG group (6.79%) was significantly lower than that in other three forests (Sum-LF:26.77%; Sum-PM: 15.22%; Sum-CL: 20.81%). The relative abundance of *Synechococcus* in Sum-CL group (12.35%) was significantly higher than that in other three forests (Sum-LF: 4.16%; Sum-CG: 2.62%; Sum-PM: 7.67%). *Merismopedia*, *Calothrix* and *Dinophysis* were abundant in all plantations (relative abundance > 4.23%). *Lobosphaera* occurred in both winter and summer and was frequently observed in all plantations.

### 3.4 Significant differences in algal community

**Figure 7:**
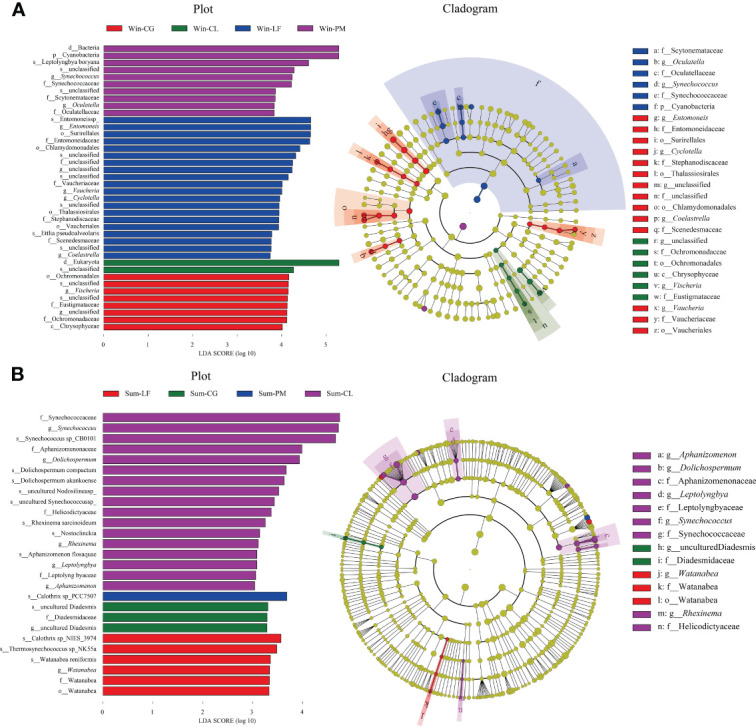
Linear discriminant analysis effect size (LEfSe) analysis of soil algal communities from winter **(A)** and summer **(B)** samples. **(A)** The left figure showing differentially abundant taxa, the histogram length represents the impact of different species [linear discriminant analysis (LDA) score > 3]; the right Cladogram showing the phylogenetic structure of the algae. The taxa with significantly different abundances of soil algae among different forest plantations identity are symbolized by colored dots. In the branching diagram of evolution, circles radiating from inside to outside represent the taxonomic level from boundary to species, and each small circle at different taxonomic levels represents a species at the taxonomic level.

LEfSe analysis further identified specific algae taxa that were differentially abundant across four different plantations (**Figure 7**).

In winter, the results suggested that the algae in four orders (i.e., Surirellales, Chlamydomonadales, Thalassiosirales, Vaucheriales), four families (i.e., Entomoneidaceae, Vaucheriaceae, Stephanodiscaceae, Scenedesmaceae), four genera (i.e., *Entomoneis*, *Vaucheria*, *Cyclotella, Coelastrella*) and two species (i.e., Entomoneis sp, Ettlia pseudoalveolaris) were significantly more abundant in *Liquidambar formosana* plantation. Algae in one class (i.e., Chrysophyceae), one order (i.e., Ochromonadales), two families (i.e., Eustigmataceae, Ochromonadaceae) and one genera (i.e., *Vischeria*) were abundant in *Cyclobalanopsis glauca* plantation. Algae in one phylum (i.e., Cyanobacteria), three families (i.e., Synechococcaceae, Scytonemataceae, Oculatellaceae), two genera (i.e., *Synechococcus, Oculatella*) and one species (i.e., Leptolyngbyaboryana) in *Pinus massoniana* plantation were noticed to be remarkably higher than others.

In summer, *Watanabea*, Thermosynechococcussp_NK55a, Watanabea reniformis, Calothrixsp_NIES_3974 were abundant in *Liquidambar formosana* plantation. Diadesmidaceae were abundant in *Cyclobalanopsis glauca* plantation. Calothrixsp_PCC7507 were abundant in *Pinus massoniana* plantation. Algae in four families (i. e., Synechococcaceae, Aphanizomenonaceae, Helicodictyaceae, Leptolyngbyaceae), five genera (*Aphanizomenon*, *Rhexinema*, *Synechococcus*, *Dolichospermum*, *Leptolyngbya*) and eight species (i.e., Synechococcus sp, Dolichospermum compactum, Dolichospermum akankoense, Rhexinema sarcinoideum, Synechococcussp_CB0101, Nosto clinckia, Nodosilinea sp, Aphanizomenon flosaquae) were abundant in *Cunninghamia lanceolata* plantation.

### 3.5 Correlation between algal community and soil physicochemical variables

**Figure 8:**
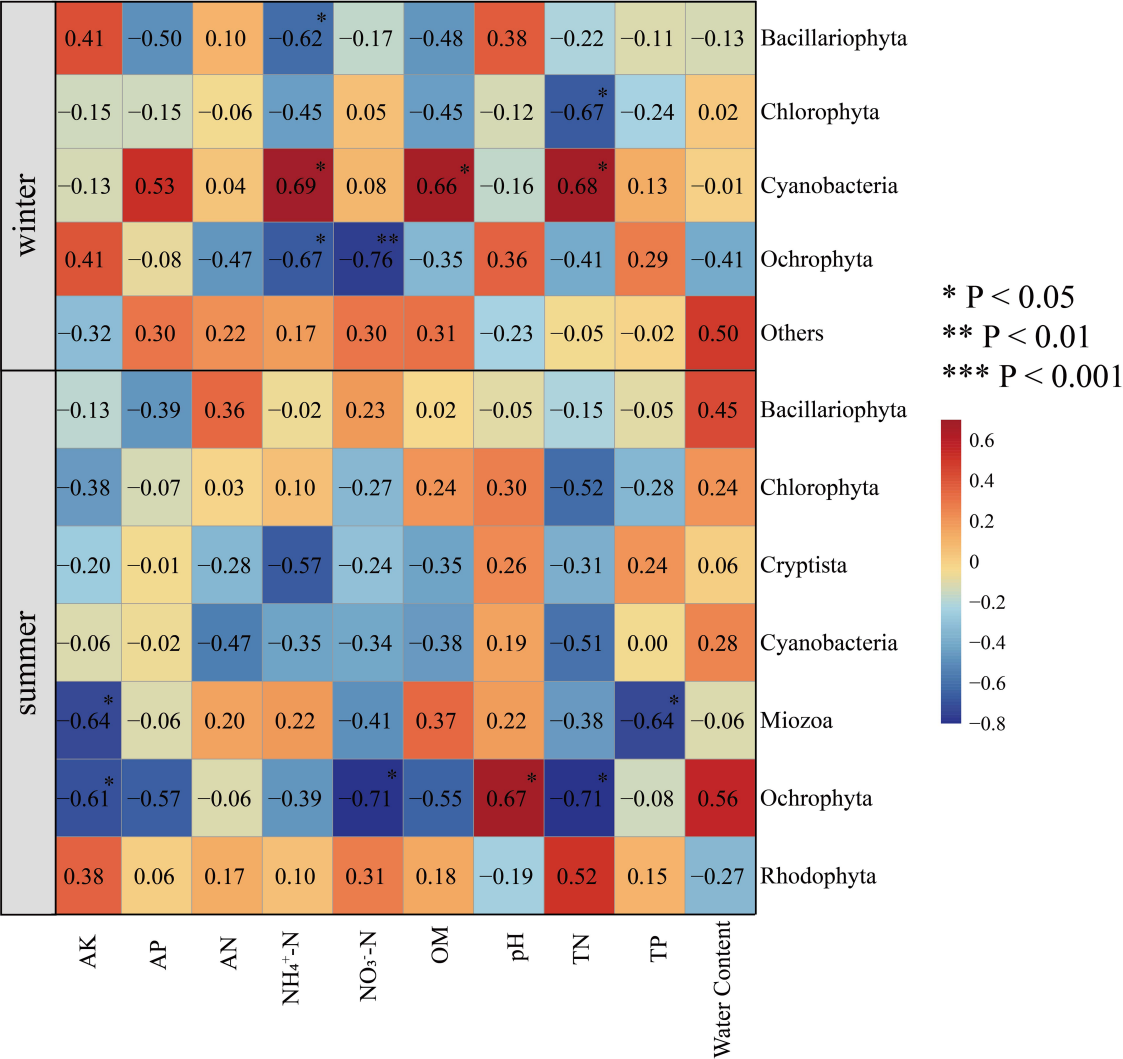
Correlation heatmap analysis of environmental factors and species. Environmental factors are on the horizontal axis, species are on the vertical axis, and color is the strength of correlation.

To study the relationship between algal community structure and environmental factors in Hule Forest Farm in different seasons, we conducted a correlation analysis between phylum abundance of algal community and environmental factors (**Figure 8**). In winter, NH_4_^+^−N (R = 0.69, *p* < 0.05), OM (R = 0.66, *p* < 0.05) and TN (R = 0.68, *p* < 0.05) was positively correlated with Cyanobacteria. There was a negatively correlation between TN and Chlorophyta (R = -0.67, p < 0.05). In summer, soil moisture content was positively correlated with Ochrophyta (R = 0.56) and Bacillariophyta (R = 0.45). The AK was negatively correlated with Ochrophyta (R = -0.61, *p* < 0.05) and Miozoa (R = -0.64, *p* < 0.05), pH was positively correlated with Ochrophyta (R = 0.67, *p* < 0.01).

**Figure 9:**
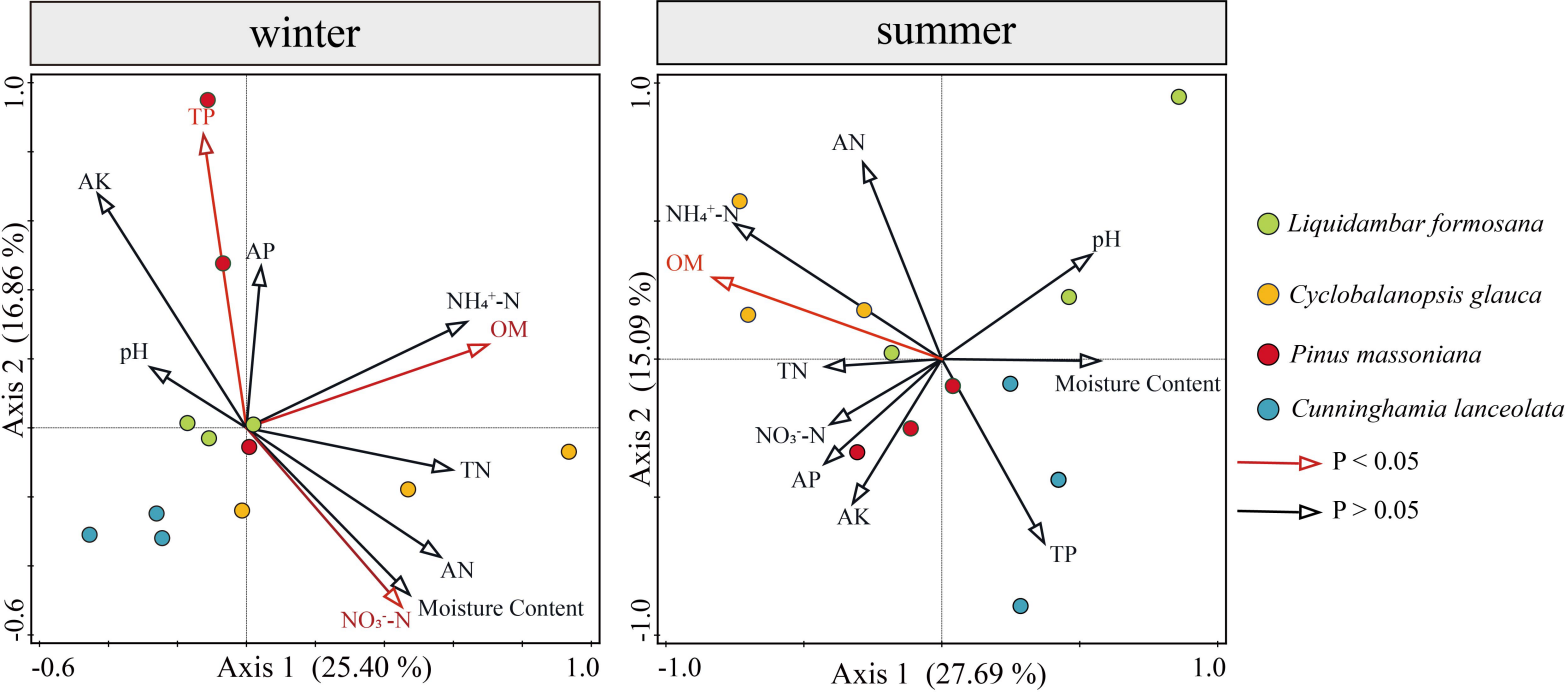
Relationships between soil physicochemical properties and algal communities in winter and summer indicated by RDA ordination plots for the first two dimensions.

Redundancy analysis (RDA analysis) was conducted with the abundance of soil algae used as the response variable, and soil physicochemical properties used as explanatory variables (environmental variables). Environmental variables in the two RDA dimensions explained 42.26% and 42.78% of the total variance in the algal community structure in winter and summer, respectively (**Figure 9**). A series of soil physicochemical factors including OM, TP and NO_3_^-^−N collectively and significantly drove the algal community structure. In winter, OM was the significant factor that provided 16.7% (p-value = 0.008, 999 Monte Carlo permutations) of the total RDA explanatory power, TP and NO_3_^-^−N were important factors as well, representing 15.4% and 12.7% of the total RDA explanatory power, respectively. In summer, OM was the most important factor, representing 21.4% (p-value = 0.004, 999 Monte Carlo permutations) of the total RDA explanatory power.

## 4 Discussion

This study showed the significant seasonal variation in the composition and relative abundance of soil algae in subtropical forests. The most abundant groups of soil algae in winter were Bacillariophyta and Chlorophyta. Cyanobacteria was the most abundant algae in summer. These findings are consistent with previous studies in other areas (Calijuri et al., 2002). Seasonal variation in soil algal community can be ascribed to the different temperature adaptation capacity of different algal groups. For example, Bacillariophyta prefer low temperature, Cyanobacteria tend to grow fast in warmer environment, and Chlorophyta are thought to be the most tolerant group to adverse soil conditions (Karsten and Holzinger, 2014). At the family level, Microcoleaceae, Oscillatoriaceae, Nostocaceae, Prasiolaceae are dominant families in winter. As the climate in our study area is relatively dry in winter, and the soil moisture content is low. Algae with distinct sheath can survive in the soil crust in dry condition, and can recover metabolic activity after receiving rainfall to improve soil productivity. In the present study, Eustigmatophyceae was most abundant in the *Pinus massoniana* plantation in winter, whcih may be due to the interactions of light and nutrients.

Both season and forest types had significant effects on soil algae in our study area (Dirborne and Ramanujam, 2017). *Liquidambar formosana* plantation has highest algal diversity due to the influence of light, which is consistent with Neustupa and Skaloud (Neustupa and Škaloud, 2008). Some algae, including Chlamydomonadales, Scenedesmaceae, Stephanodiscaceae and *Cyclotella* use to grow in shallow water, because they need strong light to grow (Calijuri et al., 2002; Reynolds et al., 2002; Padisak et al., 2008). In our study, these algae were found in the *Liquidambar formosana.* As the *Liquidambar formosana* forest had lower canopy density and thus was more conducive to the growth of these algae. The forest canopy density of *Cyclobalanopsis glauca* and *Cunninghamia lanceolata* are relatively high, and some areas even completely closed, which is more suitable for the growth of *Leptolyngbya*. Our results may suggest that the seasonal variation in algal community structure was greater than the variation across different forest plantations, but further experiments are needed to prove this.

In this study, the observed species and Shannon index of soil algae communities in four different plantations of Hule Forest Farm reached their maximum values in summer. the activity and species richness of algae increased with increasing temperature, which is consistent with previous studies (Wang et al., 2015). Temperature can directly and indirectly affect the composition and quantity of algae. On the one hand, temperature strengthens respiration by controlling the enzyme reaction of respiration, and then control the growth and reproduction of algae. On the other hand, the change of temperature would affect the dynamics of soil physicochemical properties and the nutrient cycles, which indirectly affects the growth and reproduction of algae. Many environmental factors affect the diversity of soil algal community, such as moisture content, pH, and NH_4_^+^−N (Zancan et al., 2006; Dirborne and Ramanujam, 2017; Agha et al., 2020). In the present study, spearman’s correlation analyses showed that soil moisture content was significantly and positively correlated with observed species and Shannon index (*p*< 0.01). The soil moisture exerted a great influence on the composition of soil algae. Because water plays an important role in the growth of algae, which is needed for the activities of algal filament breakup, cell division and reproductive cell germination (Nisha et al., 2007). There was a significant positive correlation between pH and algal Shannon index (*p* < 0.05), which was mainly attributed to the fact that the acidic environment would affect the photosynthetic apparatus of Cyanobacteria. Chlorophyta tend to decompose to pheophytin under mildly acidic conditions due to its acid lability (Thomas, 1973).

The diversity and distribution of soil algae are regulated by the interaction of various environmental variables, and different environmental variables have different effects on the algal community structure. Dirborne (Dirborne and Ramanujam, 2017) found that the vegetation, pH, moisture content, organic carbon and nitrogen were the main factors affecting the algal community in broadleaf sacred grove and pine forest in East Khasi Hills. Kharkongor (Kharkongor and Ramanujam, 2014) found that the algal community in forest of Meghalaya was greatly affected by sunlight, relative humidity, and rainfall. However, for Dry Mountains of Ladakh in NW Himalaya (Rehakova et al., 2011), site, altitude and vegetation type had significant influences on the distribution of soil algae. This contradiction may be partially impacted by geographical features (e.g., longitude and latitude), forest age and season.

In this study, the spearman’s correlation and RDA analysis showed that soil algal community was significantly correlated with pH, NH_4_^+^−N, NO_3_^-^−N, TP, OM and moisture content in the studied subtropical forests. Nitrogen and phosphorus are essential nutrients for algal growth, the changes of their availability can affect algal composition and diversity (Schulz et al., 2016; Liu et al., 2018). Phosphorus was a necessary component in the production of the ribosome, ATP, DNA, and RNA to maintain rapid growth, as well as an indispensable nutrient for plant growth (Delgado et al., 2017). In this study, OM was the main factor affecting soil algae in winter, because carbon sources control the heterotrophic microorganisms growth which can secrete metabolites *in vitro* to affect algae (Agnelli et al., 2021). Abundant algae play a great role in the supply of OM. Algae turnover can return organic matter to soil and provide carbon source for heterotrophic microorganisms. Therefore, soil physicochemical index can cause the change of algal community structure and function in Hule Forest Farm.

## 5 Conclusion

In this study, molecular methods were used to accurately determine the soil algal community structure of four subtropical forest plantations in winter and summer. The effects of environmental factors on soil algal community composition and diversity were also analyzed. To sum up, soil algal community structure was significantly affected by season and forest type, but the effect of season was more obvious. The diversity of soil algal community showed obvious seasonal differences, the overall diversity was higher in summer than in winter. Moreover, Chlorophyta, Bacillariophyta and Rhodophyta were mainly affected by season. In addition, we noted that OM, TP and NH_4_^+^−N were the main environmental factors affecting the distribution of algae in Hule Forest Farm. The results of the present study provide a new perspective to understand the soil algal community structure and factors influencing soil algae in forests, which can enhance the understanding of factors controlling the soil algal community structure. This would also be of great significance for evaluating the effects of afforestation with different tree species on soil algal communities.

## Data availability statement

The original contributions presented in the study are publicly available. This data can be found here: https://ncbi.nlm.nih.gov/bioproject/PRJNA948815.

## Author contributions

LW: Writing-Original Draft, Investigation, Formal analysis, visualization. QZ: Writing-Review, Editing, Supervision, Project administration. XC: Conceptualization, Methodology, Resources, Validation, Visualization, Writing - review & editing, Project administration, Funding acquisition. QS: Supervision, Writing - review & editing. XZ: Investigation, Formal analysis. YC: Writing-Review. All authors contributed to the article and approved the submitted version.
